# Supplementary material for: Machine learning-based criminal behavior analysis for enhanced digital forensics
Source: PLoS One. 2025 Oct 6;20(10):e0332802. doi: 10.1371/journal.pone.0332802 (PMC12500087; doi:10.1371/journal.pone.0332802)
Supplement: Attachment — Submitted filename: Response to reviewers.pdf [file pone.0332802.s001.pdf]

Response to Reviewers' Comments

We sincerely appreciate the time and effort you have invested in reviewing our manuscript titled **"Machine Learning-Based Criminal Behavior Analysis for Enhanced Digital Forensics."** We are grateful for your constructive feedback, which has allowed us to significantly improve the quality of our work. Below, we provide detailed responses to each of the reviewers' comments, outlining the revisions made in the revised manuscript.

Reviewer #1 Comments

1. Outdated Literature in the Review Section

Reviewer Comment: The literature review relies heavily on older sources, limiting its relevance to recent advancements in digital forensics.
Response: We have updated the literature review to incorporate **newer studies**, including **Okmi (2023)** on mobile data in crime applications, **Akbarian et al. (2023)** on AE-LSTM for anomaly detection, and **Bokolo et al. (2023)** on deep learning-assisted cybercriminal profiling. Additional relevant papers have been reviewed and cited to strengthen the theoretical framework and ensure the review reflects the latest advancements in the field.
Revision: See the revised **Literature Review section (Pages [2–5])**, which now includes a comprehensive discussion of recent studies and a **comparative analysis table (Table 1)** summarizing key research in behavioral profiling and machine learning in digital forensics.

---

2. Lack of Comparison with Related Works

Reviewer Comment: The manuscript lacks a detailed comparison with existing research, making it difficult to assess its contribution.
Response: We have now included a **comparative table (Table 1)** that compares our model with related research in behavior profiling and machine learning in digital forensics. This table highlights the **unique contributions** of our approach, such as the integration of **LSTM networks**, **Autoencoders**, and **hybrid anomaly detection techniques**, which address gaps in existing methodologies.
Revision: See **Comparison with Existing Research (Page [5])** for a detailed comparison of our model with other studies.

---

3. High False Positive Rate (18.1%)

Reviewer Comment: The model's false positive rate is relatively high, which may overwhelm investigators with irrelevant data.
Response: To reduce false positives, we have implemented a **hybrid approach** that combines **LSTM-Autoencoder** with **Random Forest** and **Isolation Forest**. This has significantly improved precision while reducing false positives to **10.7%**. Additionally, we have introduced a **dynamic thresholding mechanism** that adapts to user behavior patterns in real-time, further reducing false positives.
Revision: See **Section: Model Optimization and Performance Enhancement (Pages [8–13])** for details on the hybrid approach and dynamic thresholding.

---

4. Limited Data Scope – Focus Only on Browser Artifacts

Reviewer Comment: The model's scope is limited to browser artifacts, missing multi-platform criminal activity.
Response: We have extended our model to include **mobile logs** and **communication logs**, aligning with recent research advocating multi-device investigations. The revised model now incorporates **cross-platform tracking mechanisms** to capture user activity across multiple browsers and devices, such as **Google Chrome**, **Mozilla Firefox**, **Microsoft Edge**, and **mobile browsers**.
Revision: See **Section: Expanded Data Scope for Behavioral Profiling (Pages [6–8])** for details on the inclusion of multi-platform data.

---

5. Static Anomaly Detection Thresholds

Reviewer Comment: Using a static threshold risks missing threats or generating excessive false positives.
Response: We have replaced static thresholds with a **dynamic thresholding mechanism** that adapts to user behavior patterns in real-time. This mechanism uses the **mean and standard deviation of reconstruction errors** from the training set to dynamically adjust the anomaly detection threshold, improving sensitivity to genuine anomalies while reducing noise.
Revision: See **Section: Dynamic Thresholding Mechanism (Page [5])** for details on the implementation of dynamic thresholds.

---

6. Computational Overhead of LSTM Networks

Reviewer Comment: LSTMs are computationally demanding and may not be practical for real-time applications.
Response: We have explored **Gated Recurrent Units (GRUs)** as a lighter alternative to LSTMs, significantly reducing computational complexity while maintaining performance. Additionally, we have optimized the LSTM architecture by introducing **dropout regularization** and **attention mechanisms** to improve efficiency and reduce overfitting.
Revision: See **Section: Computational Optimization of LSTM Model (Pages [10–11])** for details on the use of GRUs and other optimizations.

---

7. Limited Cross-Browser Analysis

Reviewer Comment: The study focuses only on Chrome, Firefox, and Edge without addressing users switching between browsers.
Response: We have integrated **cross-platform tracking mechanisms** to capture user activity across multiple browsers and devices. This includes tracking **browsing history**, **search queries**, **cookies**, and **cache/temporary files** across **desktop** and **mobile browsers**. This enhancement ensures that the model can detect suspicious behavior even when users switch between browsers or devices.
Revision: See **Section: Cross-Browser and Multi-Device Tracking (Pages [6–8])** for details on the cross-platform tracking mechanisms.

Reviewer #2 Comments

8. Abstract Improvement

Reviewer Comment: The two sentences in the abstract should be merged for better readability.
Response: We have restructured the abstract to combine the two sentences into one, improving flow and clarity. The revised abstract now provides a more concise and coherent overview of the research, emphasizing the use of **LSTM networks** and **Autoencoders** for analyzing browser artifacts to detect criminal behavior.
Revision: See **Abstract section (Page [1])** for the updated abstract.

---

9. English Grammar Improvements in Related Work

Reviewer Comment: Some grammatical issues were identified in the Related Work section.
Response: We have revised and proofread the **Related Work section** for grammatical accuracy and clarity. The section has been thoroughly edited to ensure proper sentence structure, grammar, and readability.
Revision: See **Literature Review section (Pages [2–5])** for the improved version.

Additional Improvements in the Revised Manuscript

In addition to addressing the reviewers' comments, we have made the following improvements to the manuscript:

1. **Enhanced Model Architecture**: The model now includes **bidirectional LSTM layers** and **attention mechanisms** to improve context awareness and sequence reconstruction accuracy. This has significantly enhanced the model's ability to capture temporal dependencies in user behavior.
2. **Hybrid Anomaly Detection**: We have integrated **ensemble methods** (e.g., Random Forest and Isolation Forest) with the LSTM-Autoencoder to refine anomaly classification and reduce false positives. This hybrid approach has improved the model's precision and recall.
3. **Cross-Platform Behavior Profiling**: The revised model now incorporates **multi-device browsing sessions**, **cross-device search query tracking**, and **cross-platform cookie analysis** to provide a more comprehensive view of user behavior.
4. **Dynamic Thresholding**: The introduction of a **dynamic thresholding mechanism** has improved the model's ability to adapt to varying user behavior patterns, reducing false positives and enhancing anomaly detection accuracy.
5. **Performance Metrics**: The model's performance metrics have been updated, showing an **accuracy of 85.4%**, **precision of 88.1%**, **recall of 84.7%**, and an **F1-score of 86.3%**, with a reduced **false positive rate of 10.7%**.
6. **Future Research Directions**: The conclusion has been expanded to discuss potential future research, such as integrating **network traffic**, **communication logs**, and **social media activity** to provide a more holistic view of user behavior. We also explore the potential for **adversarial learning** to improve the model's resilience to evasion tactics.

Conclusion

We believe that the revisions made in response to the reviewers' comments have significantly strengthened the manuscript. The updated literature review, enhanced model architecture, and improved performance metrics demonstrate the robustness and relevance of our approach. We are confident that these changes address the reviewers' concerns and contribute to advancing the field of digital forensics.

Thank you once again for your valuable feedback. We look forward to your continued guidance and support.
