# Supplementary material for: Machine learning-based criminal behavior analysis for enhanced digital forensics
Source: PLoS One. 2025 Oct 6;20(10):e0332802. doi: 10.1371/journal.pone.0332802 (PMC12500087; doi:10.1371/journal.pone.0332802)
Supplement: Attachment — Submitted filename: Rebuttle letter.docx [file pone.0332802.s002.docx]

We sincerely appreciate the time and effort you have invested in reviewing our manuscript titled "Machine Learning-Based Criminal Behavior Analysis for Enhanced Digital Forensics." We are grateful for your constructive feedback, which has allowed us to improve the quality of our work. Below, we provide detailed responses to each of the reviewers' comments, outlining the revisions made in the revised manuscript.

### Reviewer #1

**Comment 1:**
 *The abstract could be more cohesive. Two standalone sentences on machine learning techniques can be better combined.*

**Response:**
 We agree and have revised the sentence for improved readability. The new sentence reads:

"Using advanced machine learning techniques such as Long Short-Term Memory (LSTM) networks and Autoencoders, this study focuses on detecting suspicious patterns and anomalies in browsing activity."
 This change is visible on **Page 1, Abstract section, Line 9**.

**Comment 2:**
 *The methodology section lacks detail on how artifacts were linked across devices.*

**Response:**
 We have added a comprehensive explanation of the cross-platform tracking methodology, which includes linking sessions through cookies, timestamps, and domain activity. This content is now included in **Section A: Behavior Indicators Domain Selection on Page 8–9** and **Pages 13** under “Cross-artifact correlations.”

### Reviewer #2

**Comment 1:**
 *The paper does not clarify why LSTM-based autoencoders were chosen over other models.*

**Response:**
 We have added a detailed rationale comparing LSTM-based Autoencoders with RNNs, GRUs, CAEs, and VAEs. The explanation appears in **Section C: Creation of the behavior analysis model on Page 14–15**.

**Comment 2:**
 *Include more insight into how reconstruction error is used in the model and its thresholding logic.*

**Response:**
 We expanded the explanation of reconstruction loss, including the mathematical formula used for anomaly detection and how thresholds were set based on standard deviation. These details are available on **Page 16–19**.

### Reviewer #3

**Comment 1:**
 *Quantitative results need to be compared to baseline models.*

**Response:**
 A comparative performance analysis has been added in **Section: Results on Page 20-22**, where we benchmarked our model against two baselines—Random Forest + Isolation Forest and GBM + K-Means. The performance metrics such as accuracy (85.4%), precision (88.1%), and F1-Score (86.3%) are clearly reported.

**Comment 2:**
 *Add visualizations to support evaluation.*

**Response:**
 We added three figures:

- Confusion Matrix (Fig. 3),
- Reconstruction Error Distribution (Fig. 4),
- ROC Curve (Fig. 5).
   These can be found on **Pages 21–22**.

We have made every effort to ensure the revised manuscript is improved in quality, clarity, and scientific contribution. All changes in the manuscript have been highlighted for your convenience.
