# Supplementary material for: Machine learning-based criminal behavior analysis for enhanced digital forensics
Source: PLoS One. 2025 Oct 6;20(10):e0332802. doi: 10.1371/journal.pone.0332802 (PMC12500087; doi:10.1371/journal.pone.0332802)
Supplement: Attachment — Submitted filename: Response to reviewers.docx [file pone.0332802.s003.docx]

We sincerely appreciate the time and effort you have invested in reviewing our manuscript titled "Machine Learning-Based Criminal Behavior Analysis for Enhanced Digital Forensics." We are grateful for your constructive feedback, which has allowed us to improve the quality of our work. Below, we provide detailed responses to each of the reviewers' comments, outlining the revisions made in the revised manuscript.

### Reviewer #1

**Comment 1:**
*The authors have addressed most of my previous concerns, and the manuscript has improved significantly. I appreciate the inclusion of comparative benchmarks such as the confusion matrix, ROC curve, and performance metrics. Additionally, the expanded explanation of cross-platform integration and artifact correlation adds valuable clarity and depth to the methodology section.

However, I would like to highlight one remaining issue:
While the initial concern regarding non-peer-reviewed citations appears to have been resolved, I have identified multiple citation inconsistencies in Table 1. Some entries, such as “Chand et al. (2024),” are not referenced or elaborated upon in the main text. In other cases, the authorship details or publication years differ from those cited elsewhere in the manuscript.

I strongly recommend that the authors thoroughly review and revise the citations in Table 1—as well as the rest of the manuscript—to ensure complete accuracy and consistency throughout.*

**Response:**
We sincerely thank Reviewer #1 for their thoughtful and meticulous review, particularly for taking the time to identify even the most nuanced issues within our manuscript. We deeply appreciate your constructive comments, which have significantly contributed to enhancing the overall quality and clarity of our work. Regarding the citation inconsistencies noted in Table 1 of the manuscript, we have carefully reviewed each citation one by one. As you rightly pointed out, several inconsistencies were present. These arose due to an oversight during the retyping of the manuscript in the previous revision cycle. We have since corrected these issues comprehensively. Specifically, the entry for “Chand et al. (2024)” has been removed from Table 1, as we determined that it was the mismatched citation which caused the inconsistency. In addition, all remaining minor citation inconsistencies throughout the manuscript have been addressed to ensure complete alignment between in-text references and the reference list. For your convenience, these corrections have been highlighted in the revised manuscript. We are sincerely grateful for your close attention to detail and your invaluable guidance in helping us uphold the academic integrity of our work.

For your convenience, the revised changes are mentioned below, which also marked and highlighted clearly in the document named “Revised manuscript with track changes”.

- Author name corrected in line no.124
- Research work title corrected in line no.150
- Wrong in-text citation removed in line no.154
- Mission in-text citation was added in line no.256
- Table 1 was referred within the text in line no.273
- Author name corrected in Table 1, First column, fourth row – “Fahdi et al. (2013), [14]”
- Mismatched citation “Chand et al. (2024)” was removed from Table 1, which caused the inconsistency.
- In-text citation number corrected in line no.650
- In-text citation number corrected in line no.816
- In-text citation number corrected in line no.878
- In-text citation number corrected in line no.886

We have made every effort to ensure the revised manuscript is improved in quality, clarity, and scientific contribution. All changes in the manuscript have been highlighted for your convenience.

Thank you

Yours sincerely

Deemantha Siriwardana (Corresponding Author)
